# Bayesian variable selection for high-dimensional data with an ordinal response: identifying genes associated with prognostic risk group in acute myeloid leukemia

**DOI:** 10.1186/s12859-021-04432-w

**Published:** 2021-11-02

**Authors:** Yiran Zhang, Kellie J. Archer

**Affiliations:** 1 Amgen Inc., Thousand Oaks, CA USA; 2 Division of Biostatistics, College of Public Health, The Ohio State University, Columbus, OH USA

**Keywords:** Penalized models, LASSO, Spike-and-slab, European LeukemiaNet, Bayes factor

## Abstract

**Background:**

Acute myeloid leukemia (AML) is a heterogeneous cancer of the blood, though specific recurring cytogenetic abnormalities in AML are strongly associated with attaining complete response after induction chemotherapy, remission duration, and survival. Therefore recurring cytogenetic abnormalities have been used to segregate patients into favorable, intermediate, and adverse prognostic risk groups. However, it is unclear how expression of genes is associated with these prognostic risk groups. We postulate that expression of genes monotonically associated with these prognostic risk groups may yield important insights into leukemogenesis. Therefore, in this paper we propose penalized Bayesian ordinal response models to predict prognostic risk group using gene expression data. We consider a double exponential prior, a spike-and-slab normal prior, a spike-and-slab double exponential prior, and a regression-based approach with variable inclusion indicators for modeling our high-dimensional ordinal response, prognostic risk group, and identify genes through hypothesis tests using Bayes factor.

**Results:**

Gene expression was ascertained using Affymetrix HG-U133Plus2.0 GeneChips for 97 favorable, 259 intermediate, and 97 adverse risk AML patients. When applying our penalized Bayesian ordinal response models, genes identified for model inclusion were consistent among the four different models. Additionally, the genes included in the models were biologically plausible, as most have been previously associated with either AML or other types of cancer.

**Conclusion:**

These findings demonstrate that our proposed penalized Bayesian ordinal response models are useful for performing variable selection for high-dimensional genomic data and have the potential to identify genes relevantly associated with an ordinal phenotype.

**Supplementary Information:**

The online version contains supplementary material available at 10.1186/s12859-021-04432-w.

## Background

Acute myeloid leukemia (AML) is a heterogeneous disease [1]. Cytogenetics, which is the study of chromosomes, is routinely performed in bone marrow and/or blood samples of AML patients at diagnosis. In fact, specific recurring cytogenetic abnormalities in AML strongly associate with attaining complete response after induction chemotherapy, remission duration, and survival and have therefore been used to segregate patients into favorable, intermediate, and adverse prognostic risk groups [2, 3]. These risk groups have been used to guide therapeutic decisions such as post-remission therapy [4], but still represent only a gross examination of the underlying molecular traits of AML patients. We postulate that elucidating the molecular characteristics associated with these prognostic risk groups would aid clinicians in developing a more precise understanding of this disease and potentially identify therapeutic targets. Although these prognostic risk groups are categorical, they are also ordered and therefore are an ordinal categorical response. We therefore seek to fit an ordinal response model to high-dimensional gene expression data, where the number of genes (*p*) is much greater than the sample size or number of patients (*n*).

Given the high-dimensional feature space that accompanies high-throughput gene expression assays, we desire an ordinal response method that performs automatic variable selection. While frequentist approaches to fitting penalized ordinal response models have been developed, problematically, these methods lack variable selection methods that are rooted in a hypothesis testing framework. For example, the least absolute shrinkage and selection operator (LASSO) approach [5] was previously extended to ordinal response models where the solution can be obtained through the Generalized Monotone Forward Stagewise Method [6] or coordinate descent [7]. The penalty parameter, $\lambda$, or analogously the final model, is commonly selected using information criteria (e.g., BIC, AIC), cross-validation, generalized cross-validation, or ideas based on Stein’s unbiased estimate of risk [5] and all variables having non-zero coefficients in the final model are considered important. However, a key disadvantage is that once $\lambda$ (or the final model) is selected, the parameter estimates ${\varvec{\beta }}$ are conditional on that selected value. Additionally, penalized regression models result in point estimates for the model parameters but generally lack estimates of variability, prohibiting confidence interval construction and hypothesis testing. Therefore most analysts identify variables as important predictors if they have a non-zero estimate in the selected model.

To overcome these shortcomings, herein we present four Bayesian ordinal response modeling methods that can be used to identify molecular features from high-dimensional datasets with an ordinal response. Model I is based directly on the Bayesian LASSO whereas Models II, III, and IV additionally include variable inclusion indicators. All four models permit hypothesis testing through Bayes factor which provides statistical evidence of which coefficients are or are not zero. Such approaches are relevant for identifying meaningful predictors in multivariable models, that is, to guide variable selection or identify a good subset of predictors.

## Results

### Data pre-processing

High-throughout gene expression data from the Affymetrix HGU133Plus2.0 GeneChips for 525 adult patients with de novo AML were downloaded from Gene Expression Omnibus (GSE14468) [8]. The Affymetrix Detection Call algorithm was used to determine whether probe sets were present, marginally present, or absent in each sample. The 3′:5′ ratio for *GAPDH* and the percentage of present calls for each sample was examined [9]. Subsequently, samples with any quality concerns were excluded (N = 4). To obtain probe set expression summaries, we used the robust multiarray average method [10]. We restricted our penalized Bayesian ordinal response models to the 446 patients for whom prognostic risk group was available, which included 97 favorable risk, 259 intermediate risk, and 97 with adverse risk [11]. Patients with inv(16)/t(16;16), t(8;21), or t(15;17) abnormalities, regardless of any other cytogenetic abnormality, were classified as favorable risk [8]. Patients with − 5/del(5q), − 7del(7q), t(6;9), t(9;22), 3q26 abnormality, or those complex karyotype (that is, having more than 3 abnormalities) were considered adverse risk, provided they lacked inv(16)/t(16;16), t(8;21), or t(15;17) abnormalities. All others were considered intermediate risk. We also filtered the probe sets to include only the most variable probe sets as determined by quantiles of probe set level standard deviation estimates. Prior to model fitting, probe set expression summaries were centered and scaled.

Thereafter, we fit our four proposed penalized Bayesian ordinal response models for high-dimensional covariate spaces. The prior, $\pi$, represents the proportion of important genes which can either be set to a fixed constant or assigned a hyperprior. For Models II, III, and IV, we examined four different priors where for each gene *j*, $\pi _j$ was fixed at $\pi _j = 0.50\,\forall \,j$, $\pi _j$ was fixed at $\pi _j = 0.05\,\forall \,j$, $\pi _j$ was assigned the hyperprior $\pi _j \sim {\text {Beta}}(1, 19)$, and also to increase the variance, $\pi _j$ was assigned the hyperprior $\pi _j \sim {\text {Beta}}(0.01, 0.19)$. Our four models were programmed using the “rjags” package in the R programming environment. Using the “dclone” package we ran three chains with 5000 burn-in, 5000 tuning steps, and thinned to keep every third step in the sampling process to reduce auto-correlation in our posterior samples, resulting in 9999 saved steps. Convergence was assessed using Gelman and Rubin’s potential scale reduction factor.

### Evaluation procedure for identifying important genes

We considered two methods for identifying important variables for our proposed Bayesian ordinal response models for high-dimensional data, both of which are based on hypothesis tests using Bayes factor. First, we wanted to determine whether $\beta _j$ has a non-zero effect $\forall j$. Since $\beta _j$ is continuous, it is not possible to test $\beta _j=0$ directly. Instead, we tested an interval null hypothesis [12]. Suppose $\epsilon$ is a small positive value that is close to 0. For Models I, II and III, we tested $H_{0j}: |\beta _j| \le \epsilon {\text { versus }} H_{aj}: |\beta _j| > \epsilon$. For Models II, III and IV, a variable inclusion indicator, $\gamma _j$, is included in the model. However, its incorporation into Model IV precludes our ability to test $H_{0j}: |\beta _j| \le \epsilon {\text { versus }} H_{aj}: |\beta _j| > \epsilon$ directly. Therefore, for Model IV we tested $H_{0j}: |\gamma _j\beta _j| \le \epsilon {\text { versus }} H_{aj}: |\gamma _j\beta _j| > \epsilon$ instead. Bayes factor, $B_{10}$, is defined as the ratio of posterior odds to prior odds, where the prior odds $=\frac{P(H_1)}{P(H_0)}$ and the posterior odds $= \frac{P(H_1|Data)}{P(H_0|Data)}$. The derivation of prior odds for the four models is shown in the Additional file 1 and the posterior odds was estimated empirically through MCMC posterior output [13, 14].

As previously mentioned, for Models II, III and IV a variable inclusion indicator, $\gamma _j$, is included in the model. When using the Gibbs sampler to generate sequences of $\gamma _j, j = 1,\ldots , p$ the sequences converge in distribution to the posterior distribution of $\varvec{\gamma }$ and provide relevant information for variable selection [15]. More specifically, when $\gamma _j$ is frequently one in the posterior samples, its corresponding $\beta _j$ is non-zero and therefore ${\varvec{x}}_j$ should be included in the model. When a $\gamma _j$ is more often zero in the posterior samples, its corresponding ${\varvec{x}}_j$ should be excluded from the model. Therefore, some have performed variable selection criteria based on whether the posterior mean of $\gamma _j$ is greater than 0.50 [16]. This use of the posterior probabilities of the variable inclusion indicators is an application of Bayesian Model Averaging [17]. Rather than use a threshold, we tested the hypotheses $H_{0j}: \gamma _j = 0 {\text { versus }} H_{aj}: \gamma _j = 1$ and considered the corresponding variable to be important when $H_{0j}$ is rejected. We reject $H_{0j}$ if the Bayes factor exceeded a certain threshold. The prior odds is obtained through the prior specification $\pi = t$ or $\pi \sim {\text {Beta}}(c, d)$ which is detailed for each model in the Additional file 1. The posterior odds is estimated empirically through MCMC posterior samples. To determine an appropriate threshold for Bayes factor, others previously characterized $B_{10}\in (3, 10)$ to represent substantial evidence in favor of the alternative, $B_{10}\in (10, 100)$ to represent strong evidence in favor of the alternative, and $B_{10}>100$ to represent decisive evidence in favor of the alternative [18]. In our application, we rejected $H_{0j}$ if $B_{10}>5$.

### Genes associated with acute myeloid leukemia prognostic risk group

When applying Bayes Factor to the models that fixed the prior at $\pi =0.05$ and testing $H_{0j}: \gamma _j = 0 {\text { versus }} H_{aj}: \gamma _j = 1$, Models II, III, and IV identified 8, 11, and 18 and probe sets respectively (Table 1). Similar results were obtained when using $\pi \sim {\text {Beta}}(1, 19)$ as the prior, which identified 9, 13, and 19 probe sets, respectively, or when using $\pi \sim {\text {Beta}}(0.01, 0.19)$ as the prior, which identified 8, 12, and 17 probe sets, respectively. Fewer probe sets were identified when using an uninformative prior ($\pi =0.50$), with only 4, 1, and 3 probe sets identified in Models II, III, and IV, respectively.

**Table 1:** Number of probe sets identified from Models II, III , and IV under four different priors for $\pi _j$ using Bayes Factor for $\gamma _j$ and $\beta _j$

Prior	BF	Model II	Model III	Model IV
$\pi _j=0.50$	$\gamma _j$	4	1	3
$\pi _j=0.50$	$\beta _j$ or $\beta _j\gamma _j$	4	1	2
$\pi _j=0.05$	$\gamma _j$	8	11	18
$\pi _j=0.05$	$\beta _j$ or $\beta _j\gamma _j$	4	6	20
$\pi _j\sim {\text {Beta}}(1,19)$	$\gamma _j$	9	13	19
$\pi _j\sim {\text {Beta}}(1,19)$	$\beta _j$ or $\beta \gamma _j$	4	6	20
$\pi _j\sim {\text {Beta}}(0.01,0.19)$	$\gamma _j$	8	12	17
$\pi _j\sim {\text {Beta}}(0.01,0.19)$	$\beta _j$ or $\beta \gamma _j$	4	6	18

Probe sets identified when applying Bayes Factor to test $H_{0j}: |\beta _j| \le \epsilon {\text { versus }} H_{aj}: |\beta _j| > \epsilon$ for Models II and III were always a subset of those identified when applying Bayes Factor to test $H_{0j}: \gamma _j = 0 {\text { versus }} H_{aj}: \gamma _j = 1$. Likewise probe sets identified when applying Bayes Factor to test $H_{0j}: |\gamma _j\beta _j| \le \epsilon {\text { versus }} H_{aj}: |\gamma _j\beta _j| > \epsilon$ for Model IV were always identified when testing $H_{0j}: \gamma _j = 0 {\text { versus }} H_{aj}: \gamma _j = 1$. Following previous work of others in the logistic regression setting [14], for all four models we let $\epsilon = 0.10$. When testing $H_{0j}: |\beta _j| \le \epsilon {\text { versus }} H_{aj}: |\beta _j| > \epsilon$, Model I did not identify any probe sets using Bayes Factor; in fact, the largest Bayes Factor for Model I was 1.04608. Because Model I does not include $\gamma _j$, no results are available for testing $H_{0j}: \gamma _j = 0 {\text { versus }} H_{aj}: \gamma _j = 1$. We note that when applying Bayes Factor to the $\beta _j$’s, one needs to specify $\epsilon$, which is an arbitrary choice with no suitable way of providing guidance on selecting $\epsilon$ for case applications. This threshold is not required when applying Bayes Factor to test $H_{0j}: \gamma _j = 0 {\text { versus }} H_{aj}: \gamma _j = 1$. Therefore, we prefer and recommend the $H_{0j}: \gamma _j = 0 {\text { versus }} H_{aj}: \gamma _j = 1$ testing approach.

An informative prior, when $\pi _j$ was either fixed at 0.05 or $\sim {\text {Beta}}(1,19)$ or $\sim {\text {Beta}}(0.01, 0.19)$, identified more features than an uninformative prior ($\pi _j=0.50)$. We suspect this corresponds to previous research in the frequentist setting that observed increased power under FDR control when the proportion of truly null features is accurately estimated [19]. Here the prior $\pi _j$ is an analogous quantity, reflecting the proportion of truly differentially expressed features in the dataset. For application data, the proportion of truly differentially expressed features is likely small, otherwise, a high proportion of differentially expressed genes would prove lethal to the organism. To determine if fixing $\pi _j$ versus placing a hyperprior on $\pi _j$ affected run time, we compared run times among our models and across different priors. There was not a large time difference when fixing the prior $\pi _j$ at 0.05 versus putting a Beta hyperprior on $\pi _j$ (Table 2).

**Table 2:** Run time (in hours) for each model

Prior	Model II	Model III	Model IV
$\pi _j=0.50$	6.01	5.33	3.17
$\pi _j=0.05$	5.37	5.44	2.93
$\pi _j\sim {\text {Beta}}(1,19)$	5.40	5.33	3.02
$\pi _j\sim {\text {Beta}}(0.01,0.19)$	6.58	6.53	3.54

Model I does not include the variable selection indicators hence there was no prior for $\pi _j$. The run time for Model I was 2.1739 h

We compared our results to those obtained from the BhGLM R package [20, 21]. Somewhat similar to our Model I, BhGLM includes a function bpolr that fits a Bayesian hierarchical ordered logistic regression model using a Student-t prior on $\beta _j$ rather than a double exponential prior. When this default prior was used, no probe sets were identified by bpolr.

**Table 3:** Probe sets identified by Models II, III , and IV under four different priors for $\pi _j$ using Bayes factor for testing $H_{0j}: \gamma _j = 0 {\text { versus }} H_{aj}: \gamma _j = 1$

Probe set	Symbol	Model II				Model III				Model IV			
		$\pi _j=0.50$	$\pi _j=0.05$	$\pi _j\sim {\text {Beta}}(1,19)$	$\pi _j\sim {\text {Beta}}(0.01,0.019)$	$\pi _j=0.50$	$\pi _j=0.05$	$\pi _j\sim {\text {Beta}}(1,19)$	$\pi _j\sim {\text {Beta}}(0.01,0.019)$	$\pi _j=0.50$	$\pi _j=0.05$	$\pi _j\sim {\text {Beta}}(1,19)$	$\pi _j\sim {\text {Beta}}(0.01,0.019)$
1553808_a_at	NKX2-3						X	X			X	X	X
203973_s_at	CEBPD											X	
204082_at	PBX3							X			X	X	X
204961_s_at			X	X			X	X	X		X	X	
205382_s_at	CFD										X	X	X
205844_at	VNN1											X	X
206135_at	ST18		X	X	X		X	X	X		X	X	X
208886_at	H1-0				X		X	X	X		X	X	X
208892_s_at	DUSP6										X		
210146_x_at	LILR2				X								
210755_at	HGF		X	X			X	X	X		X	X	X
210783_x_at	CLEC11A										X	X	
210933_s_at	FSCN1		X	X	X	X	X	X	X	X	X	X	X
210997_at	HGF										X	X	X
214329_x_at	TNFSF10	X	X	X	X		X	X	X	X	X	X	X
214651_s_at			X	X	X		X	X	X		X	X	X
224596_at	SLC44A1										X	X	X
224989_at									X				
225782_at	MSRB3										X	X	X
228170_at	OLIG1												X
228827_at	RUNX1T1	X	X	X	X		X	X	X		X	X	X
228904_at	HOXB3			X			X	X	X		X	X	X
242520_s_at	ARMH1		X	X	X		X	X	X	X	X	X	X
201427_s_at	SELENOP	X											
206283_s_at	TAL1	X											
223044_at	SLC40A1							X	X				

Overall, there was general consistency between the probe sets identified, with 23 unique genes mappable to 26 probe sets included in our twelve fitted models (Table 3). Because no probe sets were identified by the BhGLM R package, there is no corresponding column for BhGLM in Table 3. Ten probe sets were in common among Models II, III, and IV when considering probe sets identified by at least one of the four priors within each model. Many probe sets that were identified interrogate genes that have already been associated with AML, including *CEBPD*, *PBX3*, *DUSP6*, *LILR2*, *HGF*, *FSCN1*, *TNFSF10*, *RUNX1T1*, *HOXB3*, *TAL1*, *SLC40A1*, and *CLEC11A*. *CEBPD* is thought to be a tumor suppressor gene, given it is commonly hypermethylated in AML and thus results in low *CEBPD* expression [22]. *PBX3* is co-expressed with *HOXA9*, specifically in patients with *MLL*-rearranged AML, and these two genes coordinate synergistically in leukemogenesis [23]. *DUSP6* is a protein-tyrosine phosphatase (PTP) elevated in AML patients with *FLT3* internal tandem duplication [24]. *LILR2* interacts with *PTPN6*, another PTP which is involved in hematologic malignancies including AML [24]. Serum levels of *HGF* were higher in AML patients compared to healthy subjects, and *HGF* was prognostic for complete remission attainment, leukemia-free and overall survival in AML [25]. *FSCN1* is upregulated in several cancers and is over-expressed in AML compared to healthy controls [26]. *TNFSF10* is the gene that encodes TRAIL, a protein that induces apoptosis in tumor cells, which differed in expression levels by European Leukemia Net risk group in AML patients [27]. Further, lower levels of TRAIL conferred worse prognosis in AML patients [27]. In fact, inhibitors of histone deacetylases (HDACIs) induced expression of *TNFSF10* and hence TRAIL, demonstrating the important role of this gene in HDACI therapy in AML [28]. *RUNX1T1* is involved in the RUNX1-ETO fusion product which results from the recurrent t(8;21)(q22;q22) abnormality that is common in AML [29]. Hypomethylation of *HOXB3* was associated with increased expression in intermediate risk AML patients [30] and plays an important regulatory role, as its over-expression inhibits *FLT3*-ITD in AML patients carrying that mutation [31]. Low *TAL1* expression negatively impacts hematopoietic development and results in low myeloid production and decreased colony formation from CD34+ eythroid progenitors [32]. Low levels of *SLC40A1*, the gene that encodes the iron exporter ferroportin, has been associated with good prognosis in AML [33]. In fact, researchers previously found that *SLC40A1* had lower expression levels in patients with core-binding factor AML, who all belong to the favorable risk group [33]. *CLEC11A*, formerly known *LSLCL* with homologous protein *SCGF*, is thought to be involved in early hematopoiesis and was detected in immature neutrophils in patients with chronic and acute myeloid leukemia as well as other hematologic disorders [34]. Other probe sets were associated with genes that have been previously described as prognostic markers or implicated in other cancers (*NKX2-3*, *CFD*, *VNN1*, *ST18*, *H1-0*, *SLC44A1*, *MSRB3*, and *OLIG1*). Though the function role of *ARMH1* is unclear, NCBI’s Entrez Gene states that this gene is over-expressed in bone marrow, which may be particularly relevant in AML. Generally the expression of these genes is monotonically related to the ordinal response, cytogenetic risk group, or the expression for at least one cytogenetic risk group is well separated from the others (Additional file 2: Fig. S1). Two probe sets consistently identified by the three models, 204961_s_at and 214651_s_at, no longer map to a gene when using current annotation, though the latter was intended to interrogate *HOX9A* which is involved in leukemogenesis [23].

## Discussion

Our study differs from the initial study of this publicly available acute myeloid leukemia dataset in some fundamental ways. The initial study sought to identify genes associated with *CEBPA* mutation status, which tends to confer favorable risk [8]. Herein we were interested in identifying genes whose expression is predictive of prognostic risk group, a three-level ordinal response. The initial study used Affymetrix Microarray Suite 5 to obtain probe set expression summaries whereas we used the more commonly applied RMA method [10]. Further, the initial study used a frequentist method, Prediction Analysis of Microarrays [35], whereas we used Bayesian methods. We identified several genes that have previously been linked to AML or cancer. Nevertheless, we did not identify any of the 19 probe sets which mapped to 16 unique genes in the primary paper.

The state-of-the-art in AML diagnosis has dramatically changed over the last few decades [36]. In this study, prognostic risk groups were based on cytogenetics as defined by the Eastern Cooperative Oncology Group/Southwest Oncology group classification scheme rather than the European LeukemiaNet (ELN) risk stratification system, therefore known prognostic mutations such as *NPM1*, *FLT3*-ITD, *CEBPA*, *RUNX1*, *ASXL1*, and *TP53* were not included when defining the three ordinal classes. Since the initial study, the ELN risk stratification system was developed by consensus using an expert panel which stratified patients into four prognostic risk groups: favorable, intermediate I, intermediate II, and adverse risk [37]. An evaluation of this initial ELN standardization system in a large cohort of AML patients demonstrated these categories are associated with attainment of complete remission, disease-free survival, and overall survival in younger (< 60) and older (≥ 60) patients [38]. Due to improved genetic testing and novel discoveries regarding the importance of genetic mutations, ELN was subsequently updated and treatment decision-making guides were outlined [39]. The new ELN risk stratification system includes three ordinal levels: favorable, intermediate, and adverse. Future research to identify molecular features associated with this new ELN risk stratification system may further our understanding of AML biology and identify the prognostic relevance of molecular features.

Our penalized Bayesian ordinal response models overcome shortcomings of frequentist methods, permitting hypothesis testing through Bayes factors. Through extensive simulation studies, we previously demonstrated the superiority of Model IV, the regression-based approach with variable inclusion indicators, over two frequentist methods, ordinalgmifs and ordinalNet [40]. Others have also suggested the use of Bayesian credible intervals for variable selection [16]. Therefore, we also briefly examined the results when variables were identified as important based on equal-tailed (ET) credible intervals and Highest Posterior Density (HPD) intervals. For Models I, II and III we identified the covariates as important when their corresponding 95% equal-tailed credible or HPD intervals for $\beta _j$ did not include zero. For Model IV, we identified the covariates as important when their corresponding 95% equal-tailed credible or HPD intervals for $\gamma _j\beta _j$ did not include zero. Using 95% equal-tailed credible or HPD intervals yielded no features for Model I, and the 95% HPD intervals identified one feature for Model IV only when the prior was fixed at $\pi _j=0.05$. Somewhat similarly, for Model III, 95% equal-tailed credible intervals identified one feature when the prior fixed at $\pi _j=0.05$. However, when fitting Model II, 95% equal-tailed credible intervals identified four features when the prior fixed at $\pi _j=0.50$, two when the prior took on a Beta(0.01, 0.19) hyperprior, but one feature when the prior was either fixed at $\pi _j=0.05$ or took on a Beta(1,19) hyperprior. Some may postulate that a credible interval covering 0 indicates the predictor is not statistically reliable. However, we identified several genes associated with both AML and cancer when using Bayes Factor. Therefore, we suspect that credible and highest posterior density intervals for this gene expression dataset cover zero due to multicollinearity, which results in sign flipping of the coefficient estimates when collinear variables are included in the model. We further note that because applying Bayes Factor when testing $\beta _j$ one needs to specify $\epsilon$ which is an arbitrary choice, we prefer and advocate using Bayes Factor to test $H_{0j}: \gamma _j = 0 {\text { versus }} H_{aj}: \gamma _j = 1$ which does not require an arbitrary choice for $\epsilon$. However, we acknowledge the selecting a threshold for Bayes Factor may be a limitation, and in this paper we rejected the null hypothesis when BF > 5. This threshold can be adjusted based on recommendations from literature [18] or the maximum number of variables (e.g., genes) that researchers can validate or further explore. That is, depending upon one’s budget, time, or available samples, the threshold for Bayes Factor can be increased or decreased to adjust the number of variables identified for follow-up studies or confirmatory scientific experiments.

When assuming proportional odds, the effect of each independent variable is consistent across different levels of response categories, in other words, the slopes across the different levels of the response categories are parallel. We used the latent variable model which may not be appropriate for models without proportional odds [41]. We are working to develop penalized Bayesian stereotype logit model for high-dimensional data, which may be better suited for modeling ordinal responses that are assessed, such as cytogenetic and ELN risk group.

## Conclusions

Our penalized Bayesian ordinal response Models II, III, and IV combined with the use of Bayes Factor for testing $H_{0j}: \gamma _j = 0 {\text { versus }} H_{aj}: \gamma _j = 1$ can be used for modeling an ordinal response in the presence of a high-dimensional covariate space, such as data from high-throughput genomic assays. These identified relevant genes in our AML application data, and do not require specification of an arbitrary choice for $\epsilon$ when testing nor do they require selection of a specific value for the penalty parameter $\lambda$, because $\lambda$ is assigned a a diffuse hyperprior. Because there is similarity between resulting Models II, III, and IV but noted differences in run times, we recommend Model IV.

## Methods

Prior to introducing the four penalized Bayesian ordinal response models, we briefly review the cumulative logit model and Bayesian approaches to the cumulative logit model when $n>p$. We then review the Bayesian LASSO for continuous and dichotomous response models, then subsequently describe our four penalized Bayesian ordinal response modeling methods for $p>n$ scenarios. I is based directly on the Bayesian LASSO whereas Models II, III, and IV additionally include variable inclusion indicators.

### Cumulative logit model

Let ${\varvec{x}} = (x_{1}, x_{2} \ldots , x_{p})'$ denote a vector of observed covariates, where *p* is the number of predictors and each subject’s response, *Y*, is one of *K* ordinal categories. Let ${\varvec{\beta }} = (\beta _{1}, \beta _{2} \ldots , \beta _{p})'$ denote the vector of unknown regression parameters. Assuming proportional odds, the cumulative logit model has the form:

$$\begin{aligned} {\text {log}} \left[ \frac{Pr\left( Y\le k |{\varvec{x}}\right) }{Pr\left( Y > k | {\varvec{x}}\right) } \right] = \alpha _k - {{\varvec{\beta}}} '{\varvec{x}}, \quad k = 1, 2, \ldots ,K-1. \end{aligned}$$

where $Pr(Y\le k | {\varvec{x}})$ is the cumulative probability of the event $Y \le k$ given ${\varvec{x}}$.

Assuming the latent continuous random variable *Z*, where $Z-{{\varvec{\beta}}} '{\varvec{x}}$ follows a standard logistic distribution, the ordinal response $Y=k$ when the latent variable satisfies $\alpha _{k-1} < Z \le \alpha _k$, where the $\alpha _k$ intercepts have the constraint $-\infty = \alpha _0< \alpha _1< \alpha _2< \cdots< \alpha _{K-1} < \alpha _K = \infty$. The cumulative probability can then be represented as [42]:

$$\begin{aligned} Pr\left( Y\le k | {\varvec{x}}\right) = Pr\left( Z\le \alpha _k | {\varvec{x}}\right)&= Pr\left( Z-{{\varvec{\beta}}} '{\varvec{x}} \le \alpha _k -{\varvec{\beta}} '{\varvec{x}}\right) \\&= \frac{{\text {exp}}\left( \alpha _k - {\varvec{\beta}} '{\varvec{x}}\right) }{1+{\text {exp}}\left( \alpha _k - {\varvec{\beta}} '{\varvec{x}}\right) }, \quad k = 1, 2, \ldots ,K-1. \end{aligned}$$

#### Bayesian ordinal regression models

Albert and Chib (1993) discussed Bayesian analyses for a binary response and generalized the method to a multinomial response under ordered (i.e., ordinal) and unordered cases [43]. For the ordinal response, an underlying latent continuous distribution was assumed to be $Z_i \sim N({\varvec{\beta }}'{\varvec{x}}_i, \sigma ^2)$ for $i=1,\ldots ,n$, and modeled as a linear combination of covariates. The ordinal response was represented by imposing cut-offs to the continuous response and modeled using a cumulative probit model. They assigned a diffuse prior for regression parameters ${\varvec{\beta }}$ and cut-offs ${\varvec{\alpha }}$. They then implemented a Gibbs sampler with initial values for ${\varvec{\beta }}$ and ${\varvec{\alpha }}$ selected to be their MLEs [44, 45]. Therefore their Bayesian ordinal regression model pertained to data sets where the sample size is larger than the number of covariates.

Albert (2016) later used a uniform prior for ${\varvec{\alpha }}$, under the constraint $\alpha _2 \le \cdots \le \alpha _{K-1}$, where $\alpha _1$ was set to zero [46]. A similar uniform prior was suggested for ${\varvec{\beta }}$. The method was applied to an example data set bioChemists in the pscl R package which included 915 observations where gender, number of children aged 5 or younger, and number of articles produced by the Ph.D. mentor during the last 3 years were used to predict number of articles produced during last 3 years of Ph.D. The response variable was categorized to be ordinal with four categories, though the cut-offs for the ordinal categories were not provided. Model fitting was achieved using the MCMCoprobit function in the MCMCpack R package, which applies a hybrid Metropolis-Hastings and Gibbs algorithm under the probit link scenario.

Existing publications on proportional odds Bayesian ordinal regression models when number of observations exceeds the number of features, i.e. $p<n$, have mostly employed an underlying latent continuous variable ${\varvec{Z}}$ for outcome ${\varvec{Y}}$. The cut-off values $\alpha _1, \alpha _2, \ldots , \alpha _{K-1}$ are specified such that the ordinal outcome $Y=k$ if $\alpha _{k-1} < Z \le \alpha _k,$ for $k = 2, \ldots , K-1$. $Y=1$ is observed if $Z < \alpha _{1}$ and $Y=K$ if $Z > \alpha _{K-1}$. We note that when proportional odds are assumed, the only parameters that designate class membership are the cut-off $\alpha$’s.

### Proposed ordinal Bayesian models for high-dimensional data

#### Model I: Bayesian LASSO ordinal regression model

The seminal LASSO paper [5] briefly mentioned that the LASSO estimate can be derived as the Bayesian posterior mode when the regression parameters $\beta _j$, $j=1,\ldots ,p$, have independent double-exponential (i.e., Laplace) priors,

$$\begin{aligned} f\left( \beta _j\right) = \frac{1}{2\tau } {\text {exp}}\left( -\frac{|\beta _j|}{\tau }\right) \end{aligned}$$

where $\tau = 1/\lambda$ is the inverse of shrinkage parameter $\lambda$. Initially, the Bayesian LASSO was described for continuous [47–50] and subsequently dichotomous outcomes [14, 51]. Typically, a diffuse hyperprior for $\lambda$ [14, 50, 51] or $\lambda ^2$ [47–49] is used, which avoids the procedure of explicitly selecting a single value for the penalty term. A common choice is a Gamma(*a*, *b*) prior with small values for *a* and *b* so that the prior is diffuse and therefore non-informative [48]. It has been reported that given *a* and *b* are small (e.g. $a = 0.1, b = 0.1$), the posterior distributions are not sensitive to the choices of *a* and *b* [49] though larger values of *a* and *b* have also been used [14, 50].

Our first model (Model I) is a Bayesian LASSO ordinal regression model. Following Tibshirani [5], we assign an independent double-exponential (DE) prior to each $\beta _j$, $j = 1, \ldots p$, and extend the model from a continuous response to an ordinal response:

$$\begin{aligned}{}&{\text {log}} \left[ \frac{Pr\left( Y_i\le k |{\varvec{x}}_i\right) }{Pr\left( Y_i > k | {\varvec{x}}_i\right) } \right] = \alpha _k - \sum _{j=1}^{p}\beta _jx_{ij}, \quad {\text {for}} \ k = 1, 2, \ldots ,K-1\\&\quad \beta _j|\lambda \sim \text {DE}(0, 1/\lambda ), \quad {\text {for}} \ j = 1, \ldots , p \\&\quad \lambda \sim {\text {Gamma}}(a, b) \\&\quad \alpha _k \sim {\text {Normal}}\left( 0, \sigma ^2_{\alpha _k}\right) , \ \alpha _1< \alpha _2< \cdots < \alpha _{K-1},\quad {\text {for}} \quad k = 1, 2, \ldots , K-1 \\ \end{aligned}$$

#### Model II: spike and slab normal prior

Many Bayesian variable selection methods have been proposed in recent years. Mitchell and Beauchamp (1988) introduced the “spike and slab” prior for each regression coefficient $\beta _j, j = 1, \ldots , p$, which is a mixture of a point mass at 0 and a diffuse uniform distribution elsewhere [52]. Instead of using a probability mass at 0, George and McCulloch (1993) assigned the following prior to each $\beta _j$:

$$\begin{aligned} \beta _j | \gamma _j \sim \left( 1-\gamma _j\right) N\left( 0, \sigma _{\beta j}^2\right) + \gamma _jN\left( 0, s_j^2\sigma _{\beta j}^2\right) \end{aligned}$$

where the latent variable $\gamma _j$ takes a value of either 0 or 1 [15]. Setting $\sigma _{\beta j}^2$ to a small value leads to a small variance for $\beta _j$ such that $\beta _j$ will frequently be close to 0 when $\gamma _j = 0$. Alternatively, setting $s_j$ to a large value (e.g., $s_j > 1$) leads to a moderate or large variance for $\beta _j$ such that $\beta _j$ will frequently be non-zero when $\gamma _j = 1$. Letting

$$\begin{aligned} P\left( \gamma _j = 1\right) = 1- P\left( \gamma _j = 0\right) = \pi _j, \end{aligned}$$

then $\pi _j$ represents the prior probability that $\beta _j$ is non-zero, or the prior probability that ${\varvec{x}}_j$ should be included in the model. Two different priors for $\varvec{\gamma }$ were described. One lets each $\gamma _j$ be independent with a ${\text {Bernoulli}}(1, \pi _j)$ distribution, where fixing $\pi _j = 0.5$ is a special case. Kohn et al. (2001) discussed a more flexible approach by considering a beta hyperprior Beta(*c*, *d*) for each $\pi _j$, where $j=1, \ldots , p$ [53]. The parameters *c* and *d* can be chosen to match the desired value of mean and variance for the number of parameters that enter the model, where a smaller variance indicates a more informative hyperprior for $\pi _j$. When $c = d = 1$, $\pi _j \sim$ Uniform (0, 1), such that the hyperprior for $\pi _j$ is completely uninformative.

Our second model (Model II) assigns a prior to each $\beta _j$ similar to George and McCulloch’s (1993) “spike and slab” normal prior [15]. We assume

$$\begin{aligned} \beta _j|\gamma _j \sim \left( 1 - \gamma _j\right) \times {\text {Normal}}\left( 0, \sigma ^2_0\right) + \gamma _j \times {\text {Normal}}\left( 0, \sigma ^2_1\right) , \end{aligned}$$

where $\sigma ^2_0$ and $\sigma ^2_1$ are constant. We set $\sigma ^2_0$ to a small value and $\sigma ^2_1$ to a large value such that $\beta _j$ has a small variance when $\gamma _j =0$ and $\beta _j$ has a moderate to large variance when $\gamma _j =1$. $\sigma ^2_1$ should be selected such that the prior values for each $\beta _j$ is within a reasonable range. The model has the following formulation:

$$\begin{aligned} &{\text {log}} \left[ \frac{Pr\left( Y_i\le k |{\varvec{x}}_i\right) }{Pr\left( Y_i > k | {\varvec{x}}_i\right) } \right] = \alpha _k - \sum _{j=1}^{p}\beta _jx_{ij} , \quad {\text {for}} \ k = 1, 2, \ldots ,K-1\\&\quad \beta _j|\gamma _j \sim \left( 1 - \gamma _j\right) \times {\text {Normal}}\left( 0, \sigma ^2_0\right) + \gamma _j \times {\text {Normal}}\left( 0, \sigma ^2_1\right) , \quad {\text {for}} \ j = 1, \ldots , p\\&\quad \alpha _k \sim {\text {Normal}}\left( 0, \sigma ^2_{\alpha _k}\right) , \ \alpha _1< \alpha _2< \cdots < \alpha _{K-1},\quad {\text {for}} \quad k = 1, 2, \ldots , K-1 \\ & \quad \gamma _j \sim {\text {Bernoulli}}\left( \pi _j\right) , \quad {\text {for}} \ j = 1, \ldots , p \\&\quad \pi _j = t \ {\text {or}} \ \pi _j \sim {\text {Beta}}(c, d), \quad {\text {for}} \ j = 1, \ldots , p \end{aligned}$$

#### Model III: spike and slab LASSO prior

Yuan and Lin [54] discovered a connection between Bayesian variable selection, which introduces the binary vector $\varvec{\gamma }$, and the LASSO for a normal continuous outcome by assigning the following mixture prior to $\beta _j$:

$$\begin{aligned} \beta _j | \gamma _j \sim \left( 1-\gamma _j\right) \delta (0) + \gamma _j\text {DE}(0,\tau ), \quad j = 1, \ldots , p \end{aligned}$$

where $\tau = \frac{\lambda }{2\sigma ^2}$, $\delta (0)$ is the point mass distribution centered at zero and $DE(\tau )$ has the density $\tau {\text {exp}}(-\tau |x|)/2$. This forces $\beta _j=0$ if $\gamma _j=0$ so the model can be re-expressed under $\varvec{\gamma }$ as:

$$\begin{aligned} {\varvec{Y}}| \varvec{\gamma }, {\varvec{\beta }} \sim N\left( {\varvec{X}}_{\gamma }{\varvec{\beta }}_{\gamma }, \sigma ^2{\varvec{I}}_{n}\right) \end{aligned}$$

where ${\varvec{X}}_\gamma$ and ${\varvec{\beta }}_\gamma$ are columns of ${\varvec{X}}$ and rows of ${\varvec{\beta }}$ with corresponding $\gamma = 1$. Unlike the more widely used prior $P(\varvec{\gamma }) = \pi ^{|\varvec{\gamma }|}(1-\pi )^{1-|\varvec{\gamma }|}$ with a prespecified $\pi$, they proposed the following prior for $\varvec{\gamma }$:

$$\begin{aligned} P(\varvec{\gamma }) \propto \pi ^{|\varvec{\gamma }|}(1-\pi )^{1-|\varvec{\gamma }|} \sqrt{\text {det}\left( {\varvec{X}}'_\gamma {\varvec{X}}_\gamma \right) } \end{aligned}$$

where $|\varvec{\gamma }| = \sum \gamma _j, j = 1, \ldots , p$. Their proposed prior avoids highly correlated predictors from entering the model simultaneously. They selected the model corresponding to $\varvec{\gamma }$ that maximizes $P(\varvec{\gamma |Y})$.

The model selected by the LASSO algorithm was that having the highest posterior probability under this setting when $w =\frac{\pi }{1-\pi }\frac{\tau }{2}\sqrt{2\pi ^*\sigma ^2} = 1$ for a normal continuous outcome. To avoid confusion, the constant defining the ratio of a circle’s circumference to its diameter is represented with $\pi ^*$, whereas $\pi$ is used to denote the probability for the Bernoulli prior of $\varvec{\gamma }$. Under an orthogonal design and when $\pi =0.5$, $w=1$ is equivalent with taking $\lambda = \sqrt{\frac{8\sigma ^2}{\pi ^*}}$ and $\tau = \frac{\lambda }{2\sigma ^2}$.

The spike-and-slab LASSO assigns the following prior to each $\beta _j$:

$$\begin{aligned} \beta _j | \gamma _j \sim \left( 1-\gamma _j\right) \text {DE} \left( 0, \frac{1}{\lambda _0}\right) + \gamma _j \text {DE} \left( 0, \frac{1}{\lambda _1}\right) , \end{aligned}$$

with $\lambda _1$ small and $\lambda _0$ large and $\gamma _j = 1$ corresponding to a large $\beta _j$ effect and $\gamma _j = 0$ corresponding to a negligible or small $\beta _j$ effect [55]. The spike-and-slab LASSO has been extended to generalized linear models [20] and the Cox model [56] where a Bernoulli($\pi$) prior is assigned for each $\gamma _j$ with $\pi$ taking on either a fixed value [55] or assigned either a Beta [55] or Uniform [56] prior for $\pi$ [20].

Our third model (Model III) is an extension of Ročková and George (2018) Spike-and-Slab LASSO model [55]. We assume

$$\begin{aligned} \beta _j|\lambda ,\gamma _j \sim \left( 1 - \gamma _j\right) \times \text {DE}\left( 0, \frac{1}{\lambda _0}\right) + \gamma _j \times \text {DE}\left( 0, \frac{1}{\lambda }\right) . \end{aligned}$$

Letting $\lambda _0$ be a large positive constant (e.g. $\lambda _0 = 20$), when $\gamma _j = 0$, $\beta _j$ has small variance and clusters around 0. Instead of varying $\lambda$ at different values as Ročková and George (2018), we assign a Gamma prior $\lambda \sim {\text {Gamma}}(a, b)$. The model has the following formulation:

$$\begin{aligned} &{}{\text {log}} \left[ \frac{Pr\left( Y_i\le k |{\varvec{x}}_i\right) }{Pr\left( Y_i > k | {\varvec{x}}_i\right) } \right] = \alpha _k - \sum _{j=1}^{p}\beta _jx_{ij} , \quad {\text {for}} \ k = 1, 2, \ldots ,K-1\\ &{}\quad \beta _j|\lambda,\gamma _j \sim \left( 1 - \gamma _j\right) \times {\text {DE}}\left( 0, 1/\lambda_0\right) + \gamma _j \times {\text {DE}}\left( 0, 1/\lambda\right) , \quad {\text {for}} \ j = 1, \ldots , p\\ &{} \quad \lambda \sim {\text {Gamma}}\left(a, b\right) \\ &{}\quad \alpha _k \sim {\text {Normal}}\left( 0, \sigma ^2_{\alpha _k}\right) , \ \alpha _1< \alpha _2< \cdots < \alpha _{K-1},\quad {\text {for}} \quad k = 1, 2, \ldots , K-1 \\ &{}\quad \gamma _j \sim {\text {Bernoulli}}\left( \pi _j\right) , \quad {\text {for}} \ j = 1, \ldots , p \\ &{}\quad \pi _j = t \ {\text {or}} \ \pi _j \sim {\text {Beta}}(c, d), \quad {\text {for}} \ j = 1, \ldots , p \end{aligned}$$

#### Model IV: regression approach with variable inclusion indicator

Kuo and Mallick [57] discussed one drawback of George and McCulloch’s method, that they need to choose sophisticated tuning factors for the two variances, i.e. $\sigma _{\beta j}^2$ and $s_j^2$, in the hierarchical prior for each $\beta _j$ [57]. Instead of specifying a hierarchical model, they specified a regression model that incorporates $2^p$ submodels by including an indicator vector $\varvec{\gamma }$. Their linear regression model has the following form:

$$\begin{aligned} y_i = \sum _{j=1}^{p}\gamma _j\beta _jx_{ij} + \epsilon _i, \quad {\text {for}} \ i = 1, \ldots , n , \ j = 1, \ldots , p. \end{aligned}$$

For $j = 1, \ldots , p, \gamma _j$ is an indicator variable that takes on a value of 0 or 1. As before, when $\gamma _j = 1$, $x_j$ is included in the model. When $\gamma _j = 0$, $x_j$ is omitted from the model.

An independent Bernoulli prior, Bernoulli($\pi _j$), can be assigned to each $\gamma _j, j = 1, \ldots , p$. Kuo and Mallick [57] fixed $\pi _j$ at $0.5 \forall j$ so that the likelihood prior for each of the $2^p$ models are the same. They approximated the posterior distribution of $\varvec{\gamma }$ by means of $\varvec{\gamma }$ from the Markov chain Monte Carlo (MCMC) algorithm, and suggested that predictors having higher posterior variable inclusion indicator frequencies should be included in the model. Lykou and Ntzoufras [60] used an equivalent model for continuous outcomes and based their inferences on the posterior variable inclusion probabilities $f(\gamma _j|{\varvec{y}})$, for $j = 1, \ldots , p$ where variable *j* is selected for model inclusion if the median of $f(\gamma _j|{\varvec{y}})$ is greater than 0.5. Kuo and Mallick [57] prior for ${\varvec{\beta }}$ is equivalent to Geweke (1996) by letting $\theta _j = \gamma _j\beta _j$, for $j = 1, \ldots , p$ [58]. Then the prior for $\theta _j$ is a mixture of point mass at 0 with probability $1-\pi _j$ and normal distribution with probability $\pi _j$. This approach has been used by others [59, 60].

Our fourth model (Model IV) incorporates the Bayesian variable selection method from Kuo and Mallick [57] by including an indicator variable $\gamma _j$ for each $\beta _j$, $j = 1, \ldots , p$. We assume each $\gamma _j$ follows an independent Bernoulli distribution with probability $\pi _j$, where $\pi _j$ can be a fixed constant. Following Kohn et al. [53], we will additionally consider a more flexible approach by considering a beta hyperprior for $\pi _j$: $\pi _j \sim {\text {Beta}}(c, d)$.

$$\begin{aligned} &{\text {log}} \left[ \frac{Pr(Y_i\le k |{\varvec{x}}_i)}{Pr(Y_i > k | {\varvec{x}}_i)} \right] = \alpha _k - \sum _{j=1}^{p}\gamma _j\beta _jx_{ij} , \quad {\text {for}} \ k = 1, 2, \ldots ,K-1\\&\quad \beta _j|\lambda \sim \text {DE}(0, 1/\lambda ), \quad {\text {for}} \ j = 1, \ldots , p \\&\quad \lambda \sim {\text {Gamma}}(a, b) \\&\quad \alpha _k \sim {\text {Normal}}\left( 0, \sigma ^2_{\alpha _k}\right) , \ \alpha _1< \alpha _2< \cdots < \alpha _{K-1},\quad {\text {for}} \quad k = 1, 2, \ldots , K-1 \\&\quad \gamma _j \sim {\text {Bernoulli}}\left( \pi _j\right) , \quad {\text {for}} \ j = 1, \ldots , p \\&\quad \pi _j = t \ {\text {or}} \ \pi _j \sim {\text {Beta}}(c, d), \quad {\text {for}} \ j = 1, \ldots , p \\ \end{aligned}$$

where *t* is a constant. The priors for $\pi _j$ are specified the same way as Model II.

#### Priors

Elicitation of prior distributions is non-trivial task in Bayesian modeling. When specifying the prior variance for the $\alpha$ threshold parameters, we considered that given $\alpha _1$ and $\alpha _2$ serve as the thresholds for the latent continuous variable *Z* in determining the values of the ordinal response *Y*, both should lie within the interval [$\min (Z), \max (Z)$]. For that reason a variance $\sigma _\alpha ^2=10$ should safely encompass the range of *Z* values expected. When specifying the prior variances for $\beta _j$ for Model II, we set $\sigma _0^2=0.01$ representing a small variance given our desire for a spike at 0 when $\gamma _j=0$. We then set $\sigma _1^2=10$ representing a large variance given our desire for a slab around 0 when $\gamma _j=1$. We also performed extensive simulations which varied the values of *a* and *b* for the Gamma(*a*, *b*) prior for $\lambda$ so that the prior mean varied from 10^−2^ to 10^2^ and the prior variance $\frac{a}{b^2}$ varied from 10^−4^ to 10^4^. Our results indicate that the variable selection performance is not sensitive to the values of *a* and *b* unless they are large, for example, 100. Therefore, we used $a=b=0.1$ which has been used by others [49].

#### Code

R code for processing the application data and running all models is available at https://github.com/rennyzhang77/BayesianPenalizedCumulativeLogitModel/tree/master/BMC_Bioinformatics_2021.

## Supplementary Information

**Additional file 1.** For each model, the prior odds is derived as the prior odds isneeded for estimating Bayes Factor.

**Additional file 2: Fig. S1.** Boxplots of probe set expression by cytogenetic risk group for genes that mapped to Affymetrix probe sets identified by our penalized Bayesian ordinal response models that have been previously associated with AML. **Fig. S2.** Boxplots of probe set expression by cytogenetic risk group for genes that mapped to Affymetrix probe sets identified by our penalized Bayesian ordinal response models with no prior association with AML.

## Data Availability

High-throughout gene expression data are available from Gene Expression Omnibus accession number GSE14468.

## Abbreviations

AIC: Akaike information criteria
AML: Acute myeloid leukemia
BIC: Bayesian information criteria
DE: Double exponential
ELN: European LeukemiaNet
ET: Equal tailed
HPD: Highest posterior density
LASSO: Least absolute shrinkage and selection operator
MCMC: Markov Chain Monte Carlo
