# Supplementary material for: Bayesian variable selection for high-dimensional data with an ordinal response: identifying genes associated with prognostic risk group in acute myeloid leukemia
Source: BMC Bioinformatics. 2021 Nov 2;22:539. doi: 10.1186/s12859-021-04432-w (PMC8565083; doi:10.1186/s12859-021-04432-w)
Supplement: Supplementary file 1 — Additional file 1. For each model, the prior odds is derived as the prior odds isneeded for estimating Bayes Factor. [file 12859_2021_4432_MOESM1_ESM.pdf]

Supplemental Material: Bayesian Variable Selection for High-Dimensional Data with an Ordinal Response: Application Predicting Prognostic Risk Group in Acute Myeloid Leukemia

Yiran Zhang and Kellie J. Archer

For estimating Bayes Factor, the prior odds for each model is needed. Therefore the prior odds for Models I, II, III, and IV are outlined in turn.

Prior Odds for Model I

Our model I has the following form:

$$\log\left[\frac{Pr(Y_i \le k|\boldsymbol{x}_i)}{Pr(Y_i > k|\boldsymbol{x}_i)}\right] = \alpha_k - \sum_{j=1}^{p}\beta_j x_{ij}, \quad \text{for } k = 1, 2, ..., K-1$$

$$\beta_j|\lambda \sim \text{Double Exponential}(0, 1/\lambda), \quad \text{for } j = 1, ..., p$$

$$\lambda \sim \text{Gamma}(a, b)$$

$$\alpha_k \sim \text{Normal}(0, \sigma^2_{\alpha_k}),\ \alpha_1 < \alpha_2 < ... < \alpha_{K-1}, \quad \text{for} \quad k = 1, 2, ..., K-1$$

The marginal prior for β can be derived as:

$$\begin{aligned} f(\beta) &= \int_\lambda f(\beta|\lambda)f(\lambda)d\lambda \\ &= \int_0^\infty \frac{\lambda}{2}\exp(-\lambda|\beta|)\frac{b^a}{\Gamma(a)}\lambda^{a-1}\exp\{-b\lambda\}d\lambda \\ &= \frac{b^a}{2\Gamma(a)}\int_0^\infty \lambda^a \exp\{-\lambda(|\beta| + b)\}d\lambda \\ &= \frac{b^a\Gamma(a+1)}{2\Gamma(a)}\frac{1}{(|\beta| + b)^{a+1}}. \end{aligned}$$

Suppose $\epsilon > 0$ and is close to 0,

$$\begin{aligned} P(|\beta| \le \epsilon) &= P(-\epsilon \le \beta < 0) + P(0 \le \beta \le \epsilon) \\ &= \int_{-\epsilon}^0 \frac{b^a\Gamma(a+1)}{2\Gamma(a)}\frac{1}{(b-\beta)^{a+1}}d\beta + \int_0^\epsilon \frac{b^a\Gamma(a+1)}{2\Gamma(a)}\frac{1}{(\beta + b)^{a+1}}d\beta \\ &= \frac{b^a\Gamma(a+1)}{a\Gamma(a)}\left[\frac{1}{b^a} - \frac{1}{(b+\epsilon)^a}\right], \end{aligned}$$

$$
\begin{aligned}
P(|\beta| > \epsilon) &= P(\beta < -\epsilon) + P(\beta > \epsilon) \\
&= \int_{-\infty}^{-\epsilon} \frac{b^a \Gamma(a+1)}{2\Gamma(a)} \frac{1}{(b-\beta)^{a+1}} d\beta + \int_{\epsilon}^{\infty} \frac{b^a \Gamma(a+1)}{2\Gamma(a)} \frac{1}{(\beta+b)^{a+1}} d\beta \\
&= \frac{b^a \Gamma(a+1)}{a\Gamma(a)} \frac{1}{(b+\epsilon)^a}.
\end{aligned}
$$

The prior odds can be derived as:

$$
\frac{P(|\beta| > \epsilon)}{P(|\beta| \leq \epsilon)} = \frac{b^a}{(b+\epsilon)^a - b^a}.
$$

Prior Odds for Model II

Our model II has the following form:

$$
\begin{aligned}
&\log\left[\frac{Pr(Y_i \leq k|\boldsymbol{x}_i)}{Pr(Y_i > k|\boldsymbol{x}_i)}\right] = \alpha_k - \sum_{j=1}^{p} \beta_j x_{ij}, \quad \text{for } k = 1, 2, ..., K-1 \\
&\beta_j|\gamma_j \sim (1-\gamma_j) \times \text{Normal}(0, \sigma_0^2) + \gamma_j \times \text{Normal}(0, \sigma_1^2), \quad \text{for } j = 1, ..., p \\
&\alpha_k \sim \text{Normal}(0, \sigma_{\alpha_k}^2),\ \alpha_1 < \alpha_2 < ... < \alpha_{K-1}, \quad \text{for} \quad k = 1, 2, ..., K-1 \\
&\gamma_j \sim \text{Bernoulli}(\pi_j), \quad \text{for } j = 1, ..., p \\
&\pi_j = t \text{ or } \pi_j \sim \text{Beta}(c, d), \quad \text{for } j = 1, ..., p
\end{aligned}
$$

Setting σ_0^2 small and σ_1^2 large such that β_j has small variance if $\gamma_j = 0$ and β_j has moderate to large variance if $\gamma_j = 1$. Letting Φ denotes the CDF of standard normal distribution. If $\gamma = 1$,

$$
P(|\beta| \leq \epsilon) = \Phi\left(\frac{\epsilon}{\sigma_1}\right) - \Phi\left(-\frac{\epsilon}{\sigma_1}\right),
$$

$$
P(|\beta| > \epsilon) = 2\left[1 - \Phi\left(\frac{\epsilon}{\sigma_1}\right)\right].
$$

If $\gamma = 0$,

$$
P(|\beta| \leq \epsilon) = \Phi\left(\frac{\epsilon}{\sigma_0}\right) - \Phi\left(-\frac{\epsilon}{\sigma_0}\right),
$$

$$
P(|\beta| > \epsilon) = 2\left[1 - \Phi\left(\frac{\epsilon}{\sigma_0}\right)\right].
$$

Therefore,

$$
P(|\beta| \leq \epsilon) = P(\gamma = 0)\left[\Phi\left(\frac{\epsilon}{\sigma_0}\right) - \Phi\left(-\frac{\epsilon}{\sigma_0}\right)\right] + P(\gamma = 1)\left[\Phi\left(\frac{\epsilon}{\sigma_1}\right) - \Phi\left(-\frac{\epsilon}{\sigma_1}\right)\right],
$$

$$
P(|\beta| > \epsilon) = 2P(\gamma = 0)\left[1 - \Phi\left(\frac{\epsilon}{\sigma_0}\right)\right] + 2P(\gamma = 1)\left[1 - \Phi\left(\frac{\epsilon}{\sigma_1}\right)\right].
$$

The prior odds can be derived as:

$$\frac{P(|\beta|>\epsilon)}{P(|\beta|\le\epsilon)} = \frac{2P(\gamma=0)\Big[1-\Phi\Big(\frac{\epsilon}{\sigma_0}\Big)\Big]+2P(\gamma=1)\Big[1-\Phi\Big(\frac{\epsilon}{\sigma_1}\Big)\Big]}{P(\gamma=0)\Big[\Phi\Big(\frac{\epsilon}{\sigma_0}\Big)-\Phi\Big(-\frac{\epsilon}{\sigma_0}\Big)\Big]+P(\gamma=1)\Big[\Phi\Big(\frac{\epsilon}{\sigma_1}\Big)-\Phi\Big(-\frac{\epsilon}{\sigma_1}\Big)\Big]}.$$

Prior Odds for Model III

Our model III has the following form:

$$\begin{aligned}
&\log\Big[\frac{Pr(Y_i\le k|\boldsymbol{x}_i)}{Pr(Y_i>k|\boldsymbol{x}_i)}\Big]=\alpha_k-\sum_{j=1}^{p}\beta_j x_{ij}, \quad \text{for } k=1,2,...,K-1\\
&\beta_j|\lambda,\gamma_j \sim (1-\gamma_j)\times \text{DE}(0,1/\lambda_0)+\gamma_j\times\text{DE}(0,1/\lambda), \quad \text{for } j=1,...,p\\
&\lambda\sim\text{Gamma}(a,b)\\
&\alpha_k\sim\text{Normal}(0,\sigma^2_{\alpha_k}),\ \alpha_1<\alpha_2<...<\alpha_{K-1}, \quad \text{for} \quad k=1,2,...,K-1\\
&\gamma_j\sim\text{Bernoulli}(\pi_j), \quad \text{for } j=1,...,p\\
&\pi_j=t \text{ or } \pi_j\sim\text{Beta}(c,d), \quad \text{for } j=1,...,p
\end{aligned}$$

Letting λ_0 be a large positive number such that $1/\lambda_0$ is close to zero, for example, $1/\lambda_0 = 0.05$.

$$f(\beta|\gamma)=\begin{cases}\frac{b^a\Gamma(a+1)}{2\Gamma(a)}\frac{1}{(|\beta|+b)^{a+1}}, & \text{if } \gamma=1\\ \frac{\lambda_0}{2}\exp(-\lambda_0|\beta|), & \text{if } \gamma=0.\end{cases}$$

If $\gamma=1$,

$$P(|\beta|\le\epsilon)=\frac{b^a\Gamma(a+1)}{a\Gamma(a)}\left[\frac{1}{b^a}-\frac{1}{(b+\epsilon)^a}\right],$$

$$P(|\beta|>\epsilon)=\frac{b^a\Gamma(a+1)}{a\Gamma(a)}\frac{1}{(b+\epsilon)^a}.$$

If $\gamma=0$,

$$\begin{aligned}
P(|\beta|\le\epsilon)&=P(-\epsilon\le\beta<0)+P(0\le\beta\le\epsilon)\\
&=\int_{-\epsilon}^{0}\frac{\lambda_0}{2}\exp(-\lambda_0|\beta|)d\beta+\int_{0}^{\epsilon}\frac{\lambda_0}{2}\exp(-\lambda_0|\beta|)d\beta\\
&=1-\exp(-\lambda_0\epsilon),
\end{aligned}$$

$$\begin{aligned}
P(|\beta|>\epsilon)&=P(\beta<-\epsilon)+P(\beta>\epsilon)\\
&=\int_{-\infty}^{-\epsilon}\frac{\lambda_0}{2}\exp(-\lambda_0|\beta|)d\beta+\int_{\epsilon}^{\infty}\frac{\lambda_0}{2}\exp(-\lambda_0|\beta|)d\beta\\
&=\exp(-\lambda_0\epsilon).
\end{aligned}$$

Therefore,

$$P(|\beta|\le\epsilon)=P(\gamma=1)\frac{b^a\Gamma(a+1)}{a\Gamma(a)}\left[\frac{1}{b^a}-\frac{1}{(b+\epsilon)^a}\right]+P(\gamma=0)[1-\exp(-\lambda_0\epsilon)],$$

$$P(|\beta| > \epsilon) = P(\gamma = 1)\frac{b^a\Gamma(a+1)}{a\Gamma(a)}\frac{1}{(b+\epsilon)^a} + P(\gamma = 0)\exp(-\lambda_0\epsilon).$$

The prior odds can be derived as:

$$\frac{P(|\beta| > \epsilon)}{P(|\beta| \le \epsilon)} = \frac{P(\gamma = 1)b^a\Gamma(a+1) + P(\gamma = 0)a\Gamma(a)(b+\epsilon)^a\exp(-\lambda_0\epsilon)}{P(\gamma = 1)[(b+\epsilon)^a - b^a]\Gamma(a+1) + P(\gamma = 0)a\Gamma(a)(b+\epsilon)^a[1-\exp(-\lambda_0\epsilon)]}.$$

Prior Odds for Model IV

Our model IV has the following form:

$$\begin{aligned}
&\log\left[\frac{Pr(Y_i \le k|\boldsymbol{x}_i)}{Pr(Y_i > k|\boldsymbol{x}_i)}\right] = \alpha_k - \sum_{j=1}^{p}\gamma_j\beta_j x_{ij}, \quad \text{for } k = 1,2,...,K-1\\
&\beta_j|\lambda \sim \text{Double Exponential}(0, 1/\lambda), \quad \text{for } j = 1,...,p\\
&\lambda \sim \text{Gamma}(a,b)\\
&\alpha_k \sim \text{Normal}(0,\sigma^2_{\alpha_k}),\ \alpha_1 < \alpha_2 < ... < \alpha_{K-1}, \quad \text{for} \quad k = 1,2,...,K-1\\
&\gamma_j \sim \text{Bernoulli}(\pi_j), \quad \text{for } j = 1,...,p\\
&\pi_j = t \text{ or } \pi_j \sim \text{Beta}(c,d), \quad \text{for } j = 1,...,p
\end{aligned}$$

Assuming $\epsilon > 0$ and the priors of γ and β are independent,

$$\begin{aligned}
P(|\gamma\beta| \le \epsilon) &= P(-\epsilon \le \gamma\beta \le \epsilon)\\
&= P(\gamma\beta = 0) + P(-\epsilon \le \gamma\beta < 0) + P(0 < \gamma\beta \le \epsilon)\\
&= P(\gamma = 0) + P(\gamma = 1)P(-\epsilon \le \beta < 0) + P(\gamma = 1)P(0 < \beta \le \epsilon)\\
&= P(\gamma = 0) + P(\gamma = 1)\int_{-\epsilon}^{0}\frac{b^a\Gamma(a+1)}{2\Gamma(a)}\frac{1}{(b-\beta)^{a+1}}d\beta\\
&+ P(\gamma = 1)\int_{0}^{\epsilon}\frac{b^a\Gamma(a+1)}{2\Gamma(a)}\frac{1}{(b+\beta)^{a+1}}d\beta\\
&= P(\gamma = 0) + P(\gamma = 1)\frac{b^a\Gamma(a+1)}{a\Gamma(a)}\left[\frac{1}{b^a} - \frac{1}{(b+\epsilon)^a}\right],
\end{aligned}$$

$$\begin{aligned}
P(|\gamma\beta| > \epsilon) &= P(\gamma\beta > \epsilon) + P(\gamma\beta < -\epsilon)\\
&= P(\gamma = 1)P(\beta > \epsilon) + P(\gamma = 1)P(\beta < -\epsilon)\\
&= P(\gamma = 1)\int_{\epsilon}^{\infty}\frac{b^a\Gamma(a+1)}{2\Gamma(a)}\frac{1}{(b+\beta)^{a+1}}d\beta\\
&+ P(\gamma = 1)\int_{-\infty}^{-\epsilon}\frac{b^a\Gamma(a+1)}{2\Gamma(a)}\frac{1}{(b-\beta)^{a+1}}d\beta\\
&= P(\gamma = 1)\frac{b^a\Gamma(a+1)}{a\Gamma(a)}\frac{1}{(b+\epsilon)^a}.
\end{aligned}$$

The prior odds can be derived as:

$$\frac{P(|\gamma\beta| > \epsilon)}{P(|\gamma\beta| \le \epsilon)} = \frac{P(\gamma = 1)b^a\Gamma(a+1)}{P(\gamma = 1)((b+\epsilon)^a - b^a)\Gamma(a+1) + P(\gamma = 0)a(b+\epsilon)^a\Gamma(a)}.$$

For $j = 1, ..., p$, when $\pi_j = t$, $P(\gamma_j = 1) = t$ and $P(\gamma_j = 0) = 1 - t$. When $\pi_j \sim \text{Beta}(c, d)$,

$$\begin{aligned} P(\gamma_j = 1) &= P(\gamma_j = 1, 0 < \pi_j \leq 1) = \int_0^1 f(\gamma_j = 1|\pi_j) f(\pi_j) d\pi_j \\ &= \int_0^1 \pi_j^1 (1 - \pi_j)^0 \frac{\pi_j^{c-1}(1-\pi_j)^{d-1}}{\mathrm{B}(c,d)} d\pi_j \\ &= \frac{1}{\mathrm{B}(c,d)} \int_0^1 \pi_j^c (1-\pi_j)^{d-1} d\pi_j \\ &= \frac{\mathrm{B}(c+1,d)}{\mathrm{B}(c,d)}, \end{aligned}$$

where $\mathrm{B}(c, d) = \frac{\Gamma(c)\Gamma(d)}{\Gamma(c+d)}$. When $c = d = 1$, $P(\gamma_j = 0) = P(\gamma_j = 1) = 0.5$.
