# Supplementary material for: Bayesian variable selection for high-dimensional data with an ordinal response: identifying genes associated with prognostic risk group in acute myeloid leukemia
Source: BMC Bioinformatics. 2021 Nov 2;22:539. doi: 10.1186/s12859-021-04432-w (PMC8565083; doi:10.1186/s12859-021-04432-w)
Supplement: Supplementary file 2 — Additional file 2: Fig. S1. Boxplots of probe set expression by cytogenetic risk group for genes that mapped to Affymetrix probe sets identified by our penalized Bayesian ordinal response models that have been previously associated with AML. Fig. S2. Boxplots of probe set expression by cytogenetic risk group for genes that mapped to Affymetrix probe sets identified by our penalized Bayesian ordinal response models with no prior association with AML. [file 12859_2021_4432_MOESM2_ESM.pdf]

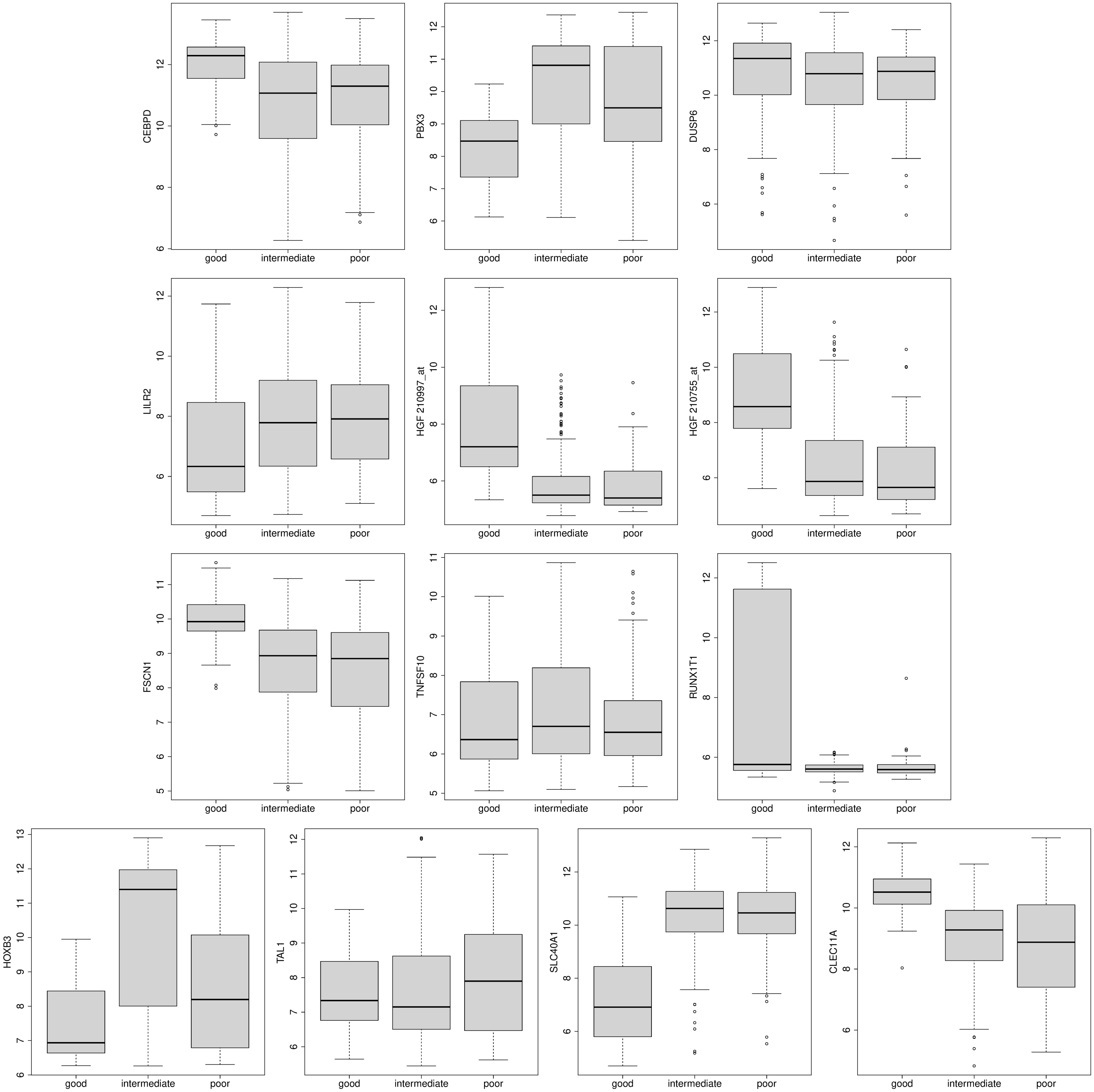

Figure 1: Boxplots of probe set expression by cytogenetic risk group for genes that mapped to Affymetrix probe sets identified by our penalized Bayesian ordinal response models that have been previously associated with AML.

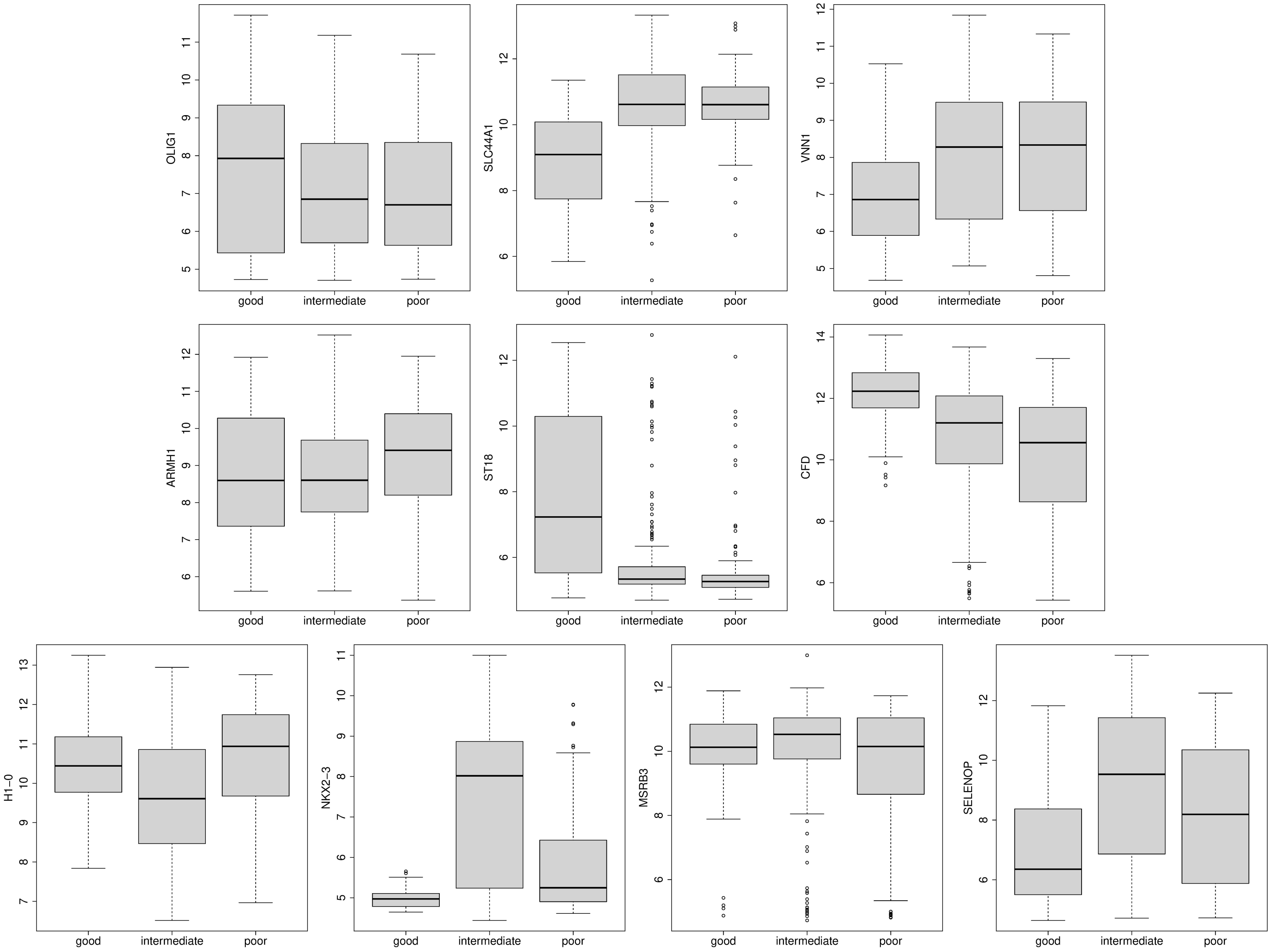

Figure 2: Boxplots of probe set expression by cytogenetic risk group for genes that mapped to Affymetrix probe sets identified by our penalized Bayesian ordinal response models not previously associated with AML.
